# Data on protein abundance alteration induced by chronic exercise in mdx mice model of Duchenne muscular dystrophy and potential modulation by apocynin and taurine

**DOI:** 10.1016/j.dib.2018.03.037

**Published:** 2018-03-19

**Authors:** Tania Gamberi, Tania Fiaschi, Elisa Valocchia, Alessandra Modesti, Paola Mantuano, Jean-Francois Rolland, Francesca Sanarica, Annamaria De Luca, Francesca Magherini

**Affiliations:** a Department of Experimental and Clinical Biomedical Sciences “Mario Serio”, University of Florence, Florence, Italy; b Section of Pharmacology, Department of Pharmacy & Drug Sciences, University of Bari “Aldo Moro”, Bari, Italy; c Axxam s.p.a., Calco, Milan, Italy

## Abstract

Here we present original data related to the research paper entitled “Proteome analysis in dystrophic mdx mouse muscle reveals a drastic alteration of Key Metabolic and Contractile Proteins after chronic exercise and the potential modulation by anti-oxidant compounds” (Gamberi et al., 2018) [1]. The dystrophin-deficient mdx mouse is the most common animal model for Duchenne muscular dystrophy. The mdx mice phenotype of the disorder is milder than in human sufferers and it can be worsened by chronic treadmill exercise. Apocynin and taurine are two antioxidant compounds proved to be beneficial on some pathology related parameters (Schröder and Schoser, 2009) [2]. This article reports the detailed proteomic data on protein abundance alterations, in tibialis anterior muscle of mdx mice, induced by chronic exercise protocol. A selected group of mdx mice was also treated with apocynin and taurine during this protocol. Detailed MS data, comparison between mdx *vs* wild type, exercised mdx *vs* wild type, and complete analysis of spot variation are provided. Furthermore, in wild type mice subjected to the same exercise protocol, the abundance of key proteins, resulted modified in exercised mdx, were analyzed by western blot.

**Specifications Table**

**Table:** 

Subject area	*Biology*
More specific subject area	*Mdx mice model for Duchenne muscular dystrophy.*
Type of data	*Table, text file, graph*
How data was acquired	*2DE gels were analyzed with Progenesis SameSpots software v4.0 (Nonlinear Dynamics, UK). MS and MSMS data were obtained with Ultraflex III MALDI- TOF/TOF mass spectrometer (Bruker Daltonics)*
Data format	*Analyzed*
Experimental factors	*Effect of chronic exercise on muscle protein abundance in mdx mice model for Duchene muscular dystrophy. Modulation by two natural compounds apocynin and taurine*
Experimental features	*Animal model. Male mdx mice divided in:*
	*-sedentary mdx (mdx) mice*
	*-exercised mdx (mdx exe) mice*
	*- mdx exercised mice treated with taurine (mdx exe tau)*
	*-mdx exercised mice treated with apocynin (mdx exe apo)*
	*-C57/BL wild-type mice exercised (wt exe) and control (wt).*
	*Age-matched male wild-type mice (C57BL/10) has been used as referring phenotype. The training protocol consisted of a 30 min running on a horizontal treadmill (Columbus Instruments, USA) at 12 m/min, twice a week for at least 4 weeks. The doses of taurine and apocynin were 1 g/kg (orally) and 38 mg/kg (1.5 mmol/l in drinking water) respectively.*
	*Proteomics: 2DE and MS were used in order to identify differences in protein abundance between groups.*
Data source location	*Male mdx mice (C57BL/10ScSn-Dmdmdx/J from Jackson Laboratories) and C57/BL wild-type (wt) mice (from Jackson Laboratories)*
Data accessibility	*Data is provided by this article*

**Value of the data**

• These data report for the first time the effect of chronic exercise protocol on protein abundance in mdx mice.
• These data can provide information about muscle damage induced by an inappropriate exercise in dystrophic patients.
• These data show the ability of taurine and apocynin to counteract some of exercise-induced protein alterations.

## 1 Data

### 1.1 MS data

97 differentially abundant spots were identified through the study published in [1]. Among these, some spots showing low Mascot (PMF) score value or discrepancy between theoretical and calculated MW or pI, were further analyzed performing peptide sequencing by tandem mass spectrometry. MS/MS analysis was carried out by using an Ultraflex III MALDI-TOF/TOF mass spectrometer (Bruker Daltonics) as described in Materials and Methods, and Table 1 reports detailed MALDI-TOF/TOF data. 12 spots show an experimental Mr different from expected. The sequence coverage of these spots is reported in Table 2. The muscle protein LIM domain-binding protein 3 (LDB3) was found in three different spots showing a Mr lower than expected. This protein belongs to Z-disc proteins whose alteration was correlated with myofibrillar myopathies [2]. Creatin kinase (Ckm) was found in six spots showing a Mr lower than expected.

**Table 1:** Differentially abundant protein spots that significantly differed between groups, identified by MALDI-TOF/TOF mass spectrometry analysis. The complete list of the proteins, identified by MALDI-TOF is reported in [1].

**Spot No**^a^			**Protein name**	**AC**^b^	**Gene Name**	**Cellular component Go term**	**Theoretical**	**Observed**	**Mascot search results**			**Peptide Sequence**^g^
							**Mr (kDa)/ pI**	**Mr (kDa)/ pI**^c^	**Score**^d^	**Matched Pept.**^e^	**Seq. coverage (%)**^f^	
**Sarcomere organization and muscle contraction**												
1		LIM domain-binding protein 3		Q9JKS4	Ldb3	Z-disc	77.6/.7.9	30.1/9.7	86	9/45	17%	[21-32] K.DFNMPLTISR.I
												[37-69] K.AAQSQLSQGDLVVAIDGVNT
												DTMTHLEAQNK.I
												[70-83] K.SASYNLSLTLQK.S
3		LIM domain-binding protein 3		Q9JKS4	Ldb3	Z-disc	77.6/.7.9	30.2/9.3	76	8/34	16%	[21-32] K.DFNMPLTISR.I
												[273-294] R.ILAQMTGTEFMQDPDEE
												ALR.R
6		Myozenin-1		Q9JK37	Myoz1	Cytoskeleton	31.4/8.6	31.7/7.9	121	15/77	67%	[42-57] R.DVMLEELSLLTNR.G
												[69-90] K.FIYENHPDVFSDSSMDHFQK.F
11		Troponin T, fast skeletal muscle		Q9QZ47	Tnnt3	Troponin complex	32.2/5.3	31.5/7.8	82	10/43	33%	[61-76] K.IPEGEKVDFDDIQK.K
												[159-175] K.ALSSMGANYSSYLAK.A
12		Troponin T, fast skeletal muscle		Q9QZ47	Tnnt3	Troponin complex	32.2/5.3	31.9/9.2	74	8/27	26%	[61-76] K.IPEGEKVDFDDIQK.K
												[159-175] K.ALSSMGANYSSYLAK.A
13		Myosin regulatory light chain 2, skeletal muscle isoform		P97457	Mylpf	Myosin complex	19/4.8	16.1/4.8	88	10/42	63%	[31-42] K.EAFTVIDQNR.D
												[41-52] R.DGIIDKEDLR.D
												[63-73] K.NEELDAMMK.E
												[92-106] K.GADPEDVITGAFK.V
16		Myosin regulatory light chain 2, skeletal muscle isoform		P97457	Mylpf	Myosin complex	19/4.8	17.1/4.9	72	6/36	37%	[31-42] K.EAFTVIDQNR.D
												[41-52] R.DGIIDKEDLR.D
												[92-106] K.GADPEDVITGAFK.V
23		Actin, alpha skeletal muscle and Actin, alpha cardiac muscle1		P68134 and P68033	Acta1 and Actc1	Cytoskeleton	42.3/5.2	42.4/5.2	72	14/32	44%	[97-116] R.VAPEEHPTLLTEAPLNPK.A
												[240-257] K.SYELPDGQVITIGNER.F
						Proteasome complex	89.9/5.1	42.4/5.2	73	8/30	27%	[25-46] R.LIVDEAINEDNSVVSLSQPK.M
		Transitional endoplasmic reticulum ATPase (mix)^¤^										
				Q01853	Vcp							
												[295-313] K.NAPAIIFIDELDAIAPK.R
		**Metabolism**										
*(Glucose metabolism)*												
30	Fructose-bisphosphate aldolase A			P05064	Aldoa	cytoplasm	39.7/8.3	30.4/7.1	60	6/25	16%	[28-43] K.GILAADESTGSIAK.R
												[111-135] K.GVVPLAGTNGETTTQGLDG
												LSER.C
												[173-201] R.YASICQQNGIVPIVEPEILPD
												GDHDLK.R
34	Triosephosphate isomerase			P17751	Tpi1	cytoplasm	32.7/5.5	25/6.7	91	8/26	34%	[56-65] K.FFVGGNWK.M
												[150-163] R.HVFGESDELIGQK.V
												[256-270] R.IIYGGSVTGATCK.E
37	Beta-enolase			P21550	Eno3	cytoplasm	47.3/6.7	46.6/6.3	95	8/22	23%	[15-29] R.GNPTVEVDLHTAK.G
												[239-254] K.VVIGMDVAASEFYR.N
*(Respiratory chain complex)*												
48	NADH dehydrogenase [ubiquinone] flavoprotein 2, mitochondrial			Q9D6J6	Ndufv2	mitochondrion	27.6/7	23.9/5.4	71	8/32	38%	[238-247] K.GPGFGVQAGL.
												[110-124] R.VYEVATFYTMYNR.K
												[41-61] R.DTPENNPDTPFDFTPENYK.R
51	ATP synthase subunit alpha, mitochondrial			Q03265	Atp5a1	mitochondrion	59.8/9.22	22.5/6.6	72	7/22	17%	[334-348] R.EAYPGDVFYLHSR.L
Energy transfert												
55	Creatine kinase M-type			P07310	Ckm	cytoplasm	43.2/6.6	24.3/6.3	61	7/20	21%	[116-131] K.GGDDLDPNYVLSSR.V
												[156-171] K.LSVEALNSLTGEFK.G
57	Creatine kinase M-type			P07310	Ckm	Cytoplasm	43.2/6.6	29/6.6	66	10/39	27%	[116-131] K.GGDDLDPNYVLSSR.V
												[156-171] K.LSVEALNSLTGEFK.G
58	Creatine kinase M-type			P07310	Ckm	Cytoplasm	43.2/6.6	29.7/6.6	61	7/35	21%	[156-171] K.LSVEALNSLTGEFK.G
												[210-215] R.DWPDAR.G
												[223-237] K.SFLVWVNEEDHLR.V
60	Creatine kinase M-type			P07310	Ckm	Cytoplasm	43.2/6.6	17.4/7.9	68	9/34	29%	[259-267] K.IEEIFKK.A
												[269-381] K.GQSIDDMIPAQK.
												[341-359] R.LGSSEVEQVQLVVDGVK.L
70	Adenylate kinase isoenzyme 1			Q9R0Y5	Ak1	Cytoplasm	21.6/5.7	21.5/5.3	58	5/20	36%	[9–22] K.IIFVVGGPGSGK.G
												[31-45] K.YGYTHLSTGDLLR.A
71	Adenylate kinase isoenzyme 1			Q9R0Y5	Ak1	Cytoplasm	21.6/5.7	22/5.5	104	11/40	55%	[9–22] K.IIFVVGGPGSGK.G
												[131-139] K.RGETSGR.V
												[139-148] R.VDDNEETIKK.R
Transport												
87	Voltage-dependent anion-selective channel protein 1			Q60932	Vdac1	Mitochondrion	32.5/8.5	29.8/8.6	74	38%	6/21	[109-123] K.LTFDSSFSPNTGK.K
												[87-107]K.WNTDNTLGTEITVEDQLAR.G
												[250-270]
												K.VNNSSLIGLGYTQTLKPGIK.L

a Spot numbers match those reported in the representative 2DE images shown in Fig. 1 and Table 1 in ref. [1]

b Accession number in Swiss-Prot/UniprotKB.

c Based on the calculation using Progenesis SameSpots 4.0 software

d MASCOT MS score (Matrix Science, London, UK; http://www.matrixscience.com). MS matching score greater than 56 was required for a significant MS hit (*p*-value<0.05).

e Number of matched peptides correspond to peptide masses matching the top hit from Ms-Fit PMF, searched peptide are also reported.

f Sequence coverage = (number of the identified residues/total number of amino acid residues in the protein sequence) x100%.

g Peptide sequence obtained by Maldi TOFTOF analysis using an Ultraflex III MALDI- TOF/TOF mass spectrometer (Bruker Daltonics).

**Table 2:** Sequence coverage (in bold) of identified proteins that show an experimental Mr different from expected.

**Spot No**^a^	**AC**^b^	**Gene Name**	c**Sequence coverage**	d**Theoretical**	e**Observed**
				**Mr (kDa)/ pI**	**Mr (kDa)/ pI**^e^
1	Q9JKS4	Ldb3	**1** MSYSVTLTGP GPWGFRLQGG K**DFNMPLTIS R**ITPGSK**AAQ SQLSQGDLVV**	77.6/.7.9	30.1/9.7
			**51 AIDGVNTDTM THLEAQNK**IK SASYNLSLTL QK**SKRPIPIS TTAPPIQSPL**		
			**101 PVIPHQK**DPA LDTNGSLATP SPSPEARASP GALEFGDTFS SSFSQTSVCS		
			**151** PLMEASGPVL PLGSPVAKAS SEGAQGSVSP KVLPGPSQPR QYNNPIGLYS		
			**201** AETLREMAQM YQMSLRGKAS GAGLLGGSLP VK**DLAVDSAS PVYQAVIKTQ**		
			**251 SKPEDEADEW AR**RSSNLQSR SFR**ILAQMTG TEYMQDPDEE ALRR**SSTPIE		
			**301** HAPVCTSQAT SPLLPASAQS PAAASPIAAS PTLATAAATH AAAASAAGPA		
			**351** ASPVENPRPQ ASAYSPAAAA SPAPSAHTSY SEGPAAPAPK PRVVTTASIR		
			**401** PSVYQPVPAS SYSPSPGANY SPTPYTPSPA PAYTPSPAPT YTPSPAPTYS		
			**451** PSPAPAYTPS PAPNYTPTPS AAYSGGPSES ASRPPWVTDD SFSQKFAPGK		
			**501** STTTVSKQTL PRGAPAYNPT GPQVTPLARG TFQRAERFPA SSRTPLCGHC		
			**551** NNVIRGPFLV AMGRSWHPEE FNCAYCKTSL ADVCFVEEQN NVYCER**CYEQ**		
			**601 FFAPICAK**CN TKIMGEVMHA LRQTWHTTCF VCAACKKPFG NSLFHMEDGE		
			**651** PYCEKDYINL FSTKCHGCDF PVEAGDKFIE ALGHTWHDTC FICAVCHVNL		
			**701** EGQPFYSKKD KPLCKKHAHA INV		
					
2	Q9JKS4	Ldb3	**1 MSYSVTLTGP GPWGFRLQGG KDFNMPLTIS R**ITPGSK**AAQ SQLSQGDLVV**	77.6/.7.9	29.6/9.7
			**51 AIDGVNTDTM THLEAQNK**IK SASYNLSLTL QKSKRPIPIS TTAPPIQSPL		
			**101** PVIPHQKDPA LDTNGSLATP SPSPEARASP GALEFGDTFS SSFSQTSVCS		
			**151** PLMEASGPVL PLGSPVAKAS SEGAQGSVSP KVLPGPSQPR QYNNPIGLYS		
			**201** AETLREMAQM YQMSLRGKAS GAGLLGGSLP VK**DLAVDSAS PVYQAVIKTQ**		
			**251 SKPEDEADEW AR**RSSNLQSR SFR**ILAQMTG TEYMQDPDEE ALRR**SSTPIE		
			**301** HAPVCTSQAT SPLLPASAQS PAAASPIAAS PTLATAAATH AAAASAAGPA		
			**351** ASPVENPRPQ ASAYSPAAAA SPAPSAHTSY SEGPAAPAPK PRVVTTASIR		
			**401** PSVYQPVPAS SYSPSPGANY SPTPYTPSPA PAYTPSPAPT YTPSPAPTYS		
			**451** PSPAPAYTPS PAPNYTPTPS AAYSGGPSES ASRPPWVTDD SFSQKFAPGK		
			**501** STTTVSKQTL PRGAPAYNPT GPQVTPLARG TFQRAERFPA SSRTPLCGHC		
			**551** NNVIRGPFLV AMGRSWHPEE FNCAYCKTSL ADVCFVEEQN NVYCERCYEQ		
			**601** FFAPICAKCN TKIMGEVMHA LRQTWHTTCF VCAACKKPFG NSLFHMEDGE		
			**651** PYCEKDYINL FSTKCHGCDF PVEAGDKFIE ALGHTWHDTC FICAVCHVNL		
			**701** EGQPFYSKKD KPLCKKHAHA INV		
					
3	Q9JKS4	Ldb3	**1** MSYSVTLTGP GPWGFRLQGG K**DFNMPLTIS R**ITPGSK**AAQ SQLSQGDLVV**	77.6/.7.9	30.2/9.3
			**51 AIDGVNTDTM THLEAQNK**IK SASYNLSLTL QK**SKRPIPIS TTAPPIQSPL**		
			**101 PVIPHQK**DPA LDTNGSLATP SPSPEARASP GALEFGDTFS SSFSQTSVCS		
			**151** PLMEASGPVL PLGSPVAKAS SEGAQGSVSP KVLPGPSQPR QYNNPIGLYS		
			**201** AETLREMAQM YQMSLRGKAS GAGLLGGSLP VK**DLAVDSAS PVYQAVIKTQ**		
			**251 SKPEDEADEW AR**RSSNLQSR SFR**ILAQMTG TEYMQDPDEE ALRR**SSTPIE		
			**301** HAPVCTSQAT SPLLPASAQS PAAASPIAAS PTLATAAATH AAAASAAGPA		
			**351** ASPVENPRPQ ASAYSPAAAA SPAPSAHTSY SEGPAAPAPK PRVVTTASIR		
			**401** PSVYQPVPAS SYSPSPGANY SPTPYTPSPA PAYTPSPAPT YTPSPAPTYS		
			**451** PSPAPAYTPS PAPNYTPTPS AAYSGGPSES ASRPPWVTDD SFSQKFAPGK		
			**501** STTTVSKQTL PRGAPAYNPT GPQVTPLARG TFQRAERFPA SSRTPLCGHC		
			**551** NNVIRGPFLV AMGRSWHPEE FNCAYCKTSL ADVCFVEEQN NVYCERCYEQ		
			**601** FFAPICAKCN TKIMGEVMHA LRQTWHTTCF VCAACKKPFG NSLFHMEDGE		
			**651** PYCEKDYINL FSTKCHGCDF PVEAGDKFIE ALGHTWHDTC FICAVCHVNL		
			**701** EGQPFYSKKD KPLCKKHAHA INV		
					
51	Q03265	Atp5a1	**1** MLSVRVAAAV ARALPRRAGL VSKNALGSSF VGARNLHASN TRLQKTGTAE	59.8/9.22	22.5/6.6
			**51** MSSILEERIL GADTSVDLEE TGRVLSIGDG IARVHGLRNV QAEEMVEFSS		
			**101** GLKGMSLNLE PDNVGVVVFG NDKLIKEGDV VKRTGAIVDV PVGEELLGRV		
			**151** VDALGNAIDG KGPIGSKTRR RVGLKAPGII PRISVREPMQ TGIKAVDSLV		
			**201** PIGRGQRELI IGDRQTGKTS IAIDTIINQK RFNDGTDEKK KLYCIYVAIG		
			**251** QKRSTVAQLV KRLTDADAMK YTIVVSATAS DAAPLQYLAP YSGCSMGEYF		
			**301** RDNGKHALII YDDLSKQAVA YRQMSLLLRR PPGR**EAYPGD VFYLHSR**LLE		
			**351** RAAKMNDSFG GGSLTALPVI ETQAGDVSAY IPTNVISITD GQIFLETELF		
			**401** YK**GIRPAINV GLSVSR**VGSA AQTRAMKQVA GTMK**LELAQY REVAAFAQFG**		
			**451 SDLDAATQQL LSR**GVRLTEL LK**QGQYSPMA IEEQVAVIYA GVR**GYLDKLE		
			**501** PSKITK**FENA FLSHVISQHQ SLLGNIR**SDG KISEQSDAKL KEIVTNFLAG		
			**551** FEP		
					
55	P07310	Ckm	**1 MPFGNTHNKF K**LNYKPQEEY PDLSKHNNHM AK**VLTPDLYN K**LRDKETPSG	43.2/6.6	24.3/6.3
			**51** FTLDDVIQTG VDNPGHPFIM TVGCVAGDEE SYTVFK**DLFD PIIQDR**HGGY		
			**101** KPTDKHKTDL NHENLK**GGDD LDPNYVLSSR** VRTGRSIK**GY TLPPHCSR**GE		
			**151** RRAVEKLSVE ALNSLTGEFK GKYYPLKSMT EQEQQQLIDD HFLFDKPVSP		
			**201** LLLASGMAR**D WPDARGIWHN DNKSFLVWVN EEDHLR**VISM EKGGNMKEVF		
			**251** RRFCVGLQKI EEIFKKAGHP FMWNEHLGYV LTCPSNLGTG LRGGVHVKLA		
			**301** NLSKHPKFEE ILTRLRLQKR GTGGVDTAAV GAVFDISNAD RLGSSEVEQV		
			**351** QLVVDGVKLM VEMEKKLEKG QSIDDMIPAQ K		
					
56	P07310	Ckm	**1 MPFGNTHNKF KLNYKPQEEY PDLSK**HNNHM AKVLTPDLYN KLRDKETPSG	43.2/6.6	28.8/6.6
			**51** FTLDDVIQTG VDNPGHPFIM TVGCVAGDEE SYTVFK**DLFD PIIQDR**HGGY		
			**101** KPTDKHKTDL NHENLKGGDD LDPNYVLSSR VR**TGRSIKGY TLPPHCSR**GE		
			**151** RRAVEK**LSVE ALNSLTGEFK** GKYYPLKSMT EQEQQQLIDD HFLFDKPVSP		
			**201** LLLASGMARD WPDARGIWHN DNK**SFLVWVN EEDHLRVISM EK**GGNMKEVF		
			**251** RRFCVGLQKI EEIFKKAGHP FMWNEHLGYV LTCPSNLGTG LRGGVHVKLA		
			**301** NLSKHPKFEE ILTRLRLQKR GTGGVDTAAV GAVFDISNAD RLGSSEVEQV		
			**351** QLVVDGVKLM VEMEKKLEKG QSIDDMIPAQ K		
					
57	P07310	Ckm	**1 MPFGNTHNKF KLNYKPQEEY PDLSK**HNNHM AKVLTPDLYN KLRDKETPSG	43.2/6.6	29/6.6
			**51** FTLDDVIQTG VDNPGHPFIM TVGCVAGDEE SYTVFK**DLFD PIIQDRHGGY**		
			**101 KPTDKHK**TDL NHENLKGGDD LDPNYVLSSR VR**TGRSIKGY TLPPHCSR**GE		
			**151** RRAVEK**LSVE ALNSLTGEFK** GKYYPLKSMT EQEQQQLIDD HFLFDKPVSP		
			**201** LLLASGMAR**D WPDAR**GIWHN DNK**SFLVWVN EEDHLRVISM EKGGNMK**EVF		
			**251** RRFCVGLQKI EEIFKKAGHP FMWNEHLGYV LTCPSNLGTG LRGGVHVKLA		
			**301** NLSKHPKFEE ILTRLRLQKR GTGGVDTAAV GAVFDISNAD RLGSSEVEQV		
			**351** QLVVDGVKLM VEMEKKLEKG QSIDDMIPAQ K		
					
58	P07310	Ckm	**1 MPFGNTHNKF KLNYKPQEEY PDLSK**HNNHM AK**VLTPDLYN K**LRDKETPSG	43.2/6.6	29.7/6.6
			**51** FTLDDVIQTG VDNPGHPFIM TVGCVAGDEE SYTVFK**DLFD PIIQDR**HGGY		
			**101** KPTDKHKTDL NHENLKGGDD LDPNYVLSSR VRTGRSIK**GY TLPPHCSR**GE		
			**151** RRAVEK**LSVE ALNSLTGEFK** GKYYPLKSMT EQEQQQLIDD HFLFDKPVSP		
			**201** LLLASGMARD WPDARGIWHN DNK**SFLVWVN EEDHLR**VISM EKGGNMKEVF		
			**251** RRFCVGLQKI EEIFKKAGHP FMWNEHLGYV LTCPSNLGTG LRGGVHVKLA		
			**301** NLSKHPKFEE ILTRLRLQKR GTGGVDTAAV GAVFDISNAD RLGSSEVEQV		
			**351** QLVVDGVKLM VEMEKKLEKG QSIDDMIPAQ K		
					
59	P07310	Ckm	**1 MPFGNTHNKF KLNYKPQEEY PDLSK**HNNHM AKVLTPDLYN KLRDKETPSG	43.2/6.6	24.4/6.5
			**51** FTLDDVIQTG VDNPGHPFIM TVGCVAGDEE SYTVFK**DLFD PIIQDR**HGGY		
			**101** KPTDKHKTDL NHENLKGGDD LDPNYVLSSR VRTGRSIK**GY TLPPHCSR**GE		
			**151** RRAVEK**LSVE ALNSLTGEFK** GKYYPLKSMT EQEQQQLIDD HFLFDKPVSP		
			**201** LLLASGMARD WPDARGIWHN DNKSFLVWVN EEDHLR**VISM EKGGNMK**EVF		
			**251** RRFCVGLQKI EEIFKKAGHP FMWNEHLGYV LTCPSNLGTG LRGGVHVKLA		
			**301** NLSKHPKFEE ILTRLRLQKR GTGGVDTAAV GAVFDISNAD RLGSSEVEQV		
			**351** QLVVDGVKLM VEMEKKLEKG QSIDDMIPAQ K		
					
60	P07310	Ckm	**1 MPFGNTHNKF K**LNYKPQEEY PDLSKHNNHM AK**VLTPDLYN K**LRDKETPSG	43.2/6.6	17.4/7.9
			**51** FTLDDVIQTG VDNPGHPFIM TVGCVAGDEE SYTVFKDLFD PIIQDRHGGY		
			**101** KPTDKHKTDL NHENLKGGDD LDPNYVLSSR VRTGRSIKGY TLPPHCSRGE		
			**151** RRAVEKLSVE ALNSLTGEFK GKYYPLKSMT EQEQQQLIDD HFLFDKPVSP		
			**201** LLLASGMAR**D WPDAR**GIWHN DNK**SFLVWVN EEDHLR**VISM EKGGNMKEVF		
			**251** RRFCVGLQKI EEIFKK**AGHP FMWNEHLGYV LTCPSNLGTG LR**GGVHVKLA		
			**301** NLSKHPK**FEE ILTR**LRLQK**R GTGGVDTAAV GAVFDISNAD RLGSSEVEQV**		
			**351 QLVVDGVK**LM VEMEKKLEKG QSIDDMIPAQ K		
					
90	Q9R1S8	Capn7	**1** MDASALERDA VQFARLAVQR DHEGRYSEAV FYYK**EAAQAL IYAEMAGSSL**	93.3/8.1	17.6/10.3
			**51 ERIQEK**INEY LERVQALHSA VQSK**STDPLK** SKHQLDLER**A HFLVTQAFDE**		
			**101 DEKGNVEDAI ELYTEAVELC LK**TSSETADK TLQNKLKQLA RQALDRAEAL		
			**151** SEPLTKPFCK LKSANMK**TKT PPVR**THFPLG PNPFVEKPQA FISPQSCDAQ		
			**201** GQKYTAEEIE VLRTTSKING VEYVPFMSVD LRERFAYPMP FCDRLGKLPL		
			**251** SPKQKTTFSK WVRPEDLTNN PTMIYTVSSF SIKQTIVSDC SFVASLAISA		
			**301** AYERRFNKKL ITSIIYPQNK DGEPEYNPCG KYMVKLHLNG VPRKVIIDDQ		
			**351** LPVDHKGELL CSYSNNKSEL WVSLIEK**AYM KVMGGYDFPG SNSNIDLHAL**		
			**401 TGWIPERIAM HSDSQTFSKD NSFR**MLYQRF HKGDVLITAS TGVMTEAEGE		
			**451** KWGLVPTHAY AVLDIREFKG LRFIQLKNPW SHLRWKGRYS ENDVKNWTPE		
			**501** LQKYLNFDPR TAQKIDNGIF WISWDDLCQY YDVVYLSWNP ALFKESTCIH		
			**551** STWDAKQGPV K**DAYSLANNP QYKLEVQCPQ GGAAVWVLLS R**HITDKDDFA		
			**601** NNREFITMVV YKTDGKKVYY PADPPPYIDG IRINSPHYLT KIKLTTPGTH		
			**651** TFTLVVSQYE KQNTIHYTVR VYSACSFTFS KIPSPYTLSK RINGKWSGQS		
			**701** AGGCGNFQET HKNNPIYQFH IDKTGPLLIE LRGPR**QYSVG FEVVAVSIMG**		
			**751 DPGPHGFQRK** SSGDYRCGFC YLELENIPAG IFNIIPSTFL PK**QEGPFFLD**		
			**801 FNSTVPIK**TT QLQ		
					
96	Q80XQ2	Tbc1d5	**1** MYKSVSETRH PLQSEEQEVG IDPLFSYSNK TRGDLSQNGR GSNSTLDTEG	92.3/6.3	35.1/6.6
			**51** TFNSYMKEWE ELFVNNNYLA TVRQKGINGQ LRSSRFRSIC WKLFLCVLPQ		
			**101** DKSQWISKIK ELRAWYSSIK EIHITNPRKA AGQQDLMINN PLSQDEGSLW		
			**151** NK**FFQDKELR SMIEQDVK**RT FPEMQFFQQE NVRKILTDVL FCYARENEQL		
			**201** LYKQGMHELL APIIFTLHCD HQAFLHASES AQPSEEMKTL LNPEYLEHDA		
			**251** YAMFSQLMET AEPWFSTFEH DGQKGKETLM APIPFARPQD LGPTVAIVTK		
			**301** VNQIQDHLLK KHDIELYMHL NRLEIAPQIY GLRWVRLLFG REFPLQDLLV		
			**351** VWDALFADSL NLSLVDYVFT AMLLYIRDAL ISSNYQTCLG LLMHYPIIGD		
			**401** IHSLILKALF LRDPKRNPRP ATYQFHPNLD YYKARGADLM NKSRTNARGA		
			**451** PLNIHKVSNS LINFGRK**LIS PASAPGSMGG PVPGNNSSSS FSAAIPTR**TS		
			**501** TEAPRHHLLQ QQQQQQHQQQ QQQQPQQQQQ QHQQQQQQQR LMKSESMPVQ		
			**551** LNKGQSSKTI SSSPSIESLP GGREFTGSPP PSATKKDSFF SNIAR**SRSHS**		
			**601 K**TMGRKESEE ELEAQISFLQ GQLNDLDAMC K**YCAKVMDMH LVNIQDVVLQ**		
			**651 ENLEK**EDQIL VSLAGLKQIK DILKGSLRFN QSQLEAGENE QITIADDHYC		
			**701** SSGQDQGSQV PRAAKQASSE MPGCTGGTTP DDFILVSKED EGHRARGAFS		
			**751** GQAQPLLTLR STSGK**SRAPA CSPLLFSDPL MGPASASASS SNPSSSPDDD**		
			**801 SSK**ESGFTIV SPLDI		

a Spot numbers match those reported in the representative 2DE images shown in Fig. 1 and Table 1 in ref. [1]

b Accession number in Swiss-Prot/UniprotKB.

c Sequence coverage refers to the identified peptides of the protein sequence (bold letters).

d Theoretical molecular mass (Mr) and isoelectric point (pI) according to protein sequence.

e Molecular mass (Mr) and isoelectric point (pI) based on the calculation using software Progenesis SameSpots

### 1.2 Apocynin and taurine modulate the effect of exercise on mdx mice muscle protein abundance

Fig. 1 reports 97 histograms representing the spot abundance, in each group analysed (mdx, mdx exe, mdx exe tau, mdx exe apo) evaluated by gel image analysis with ProgenesisSame Spot. Proteins are divided in categories according to their GO biological process. Protein spot abundance in wt mice was also evaluated as referring phenotype. Fig. 2 summarizes the modulatory effects of taurine and apocynin.

**Fig. 1:**
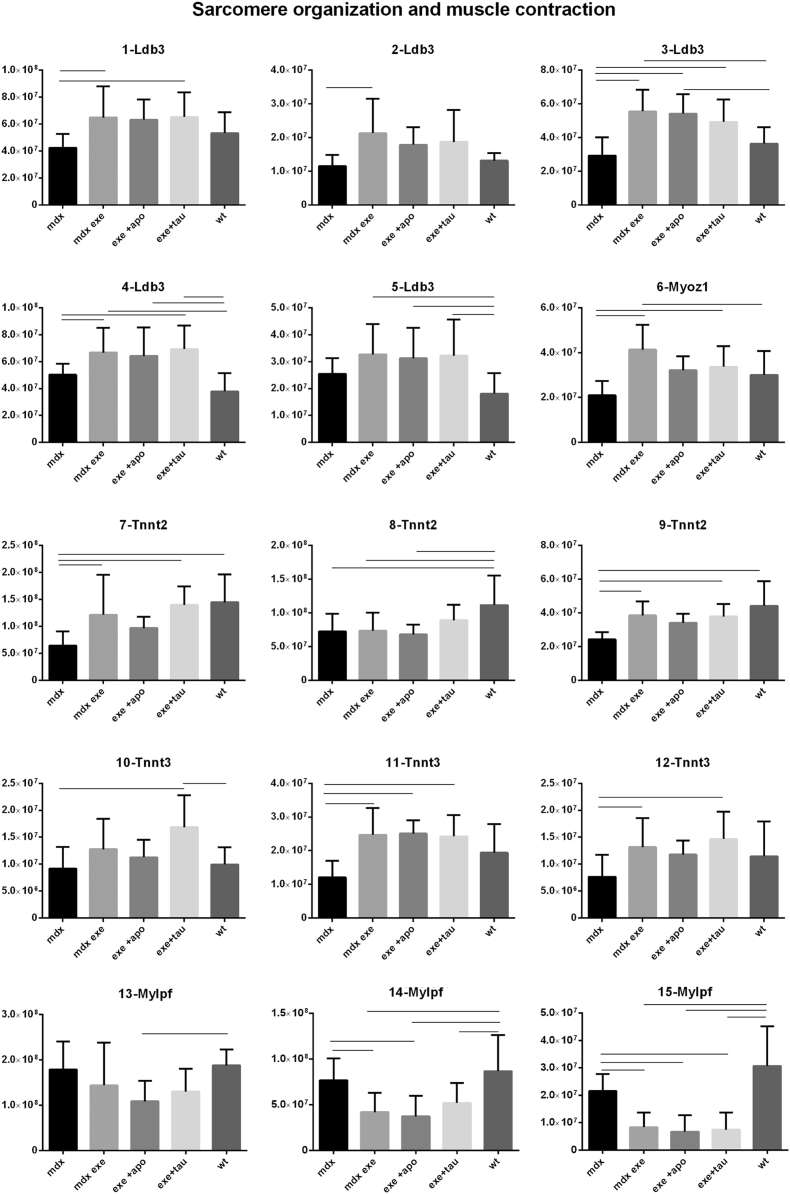

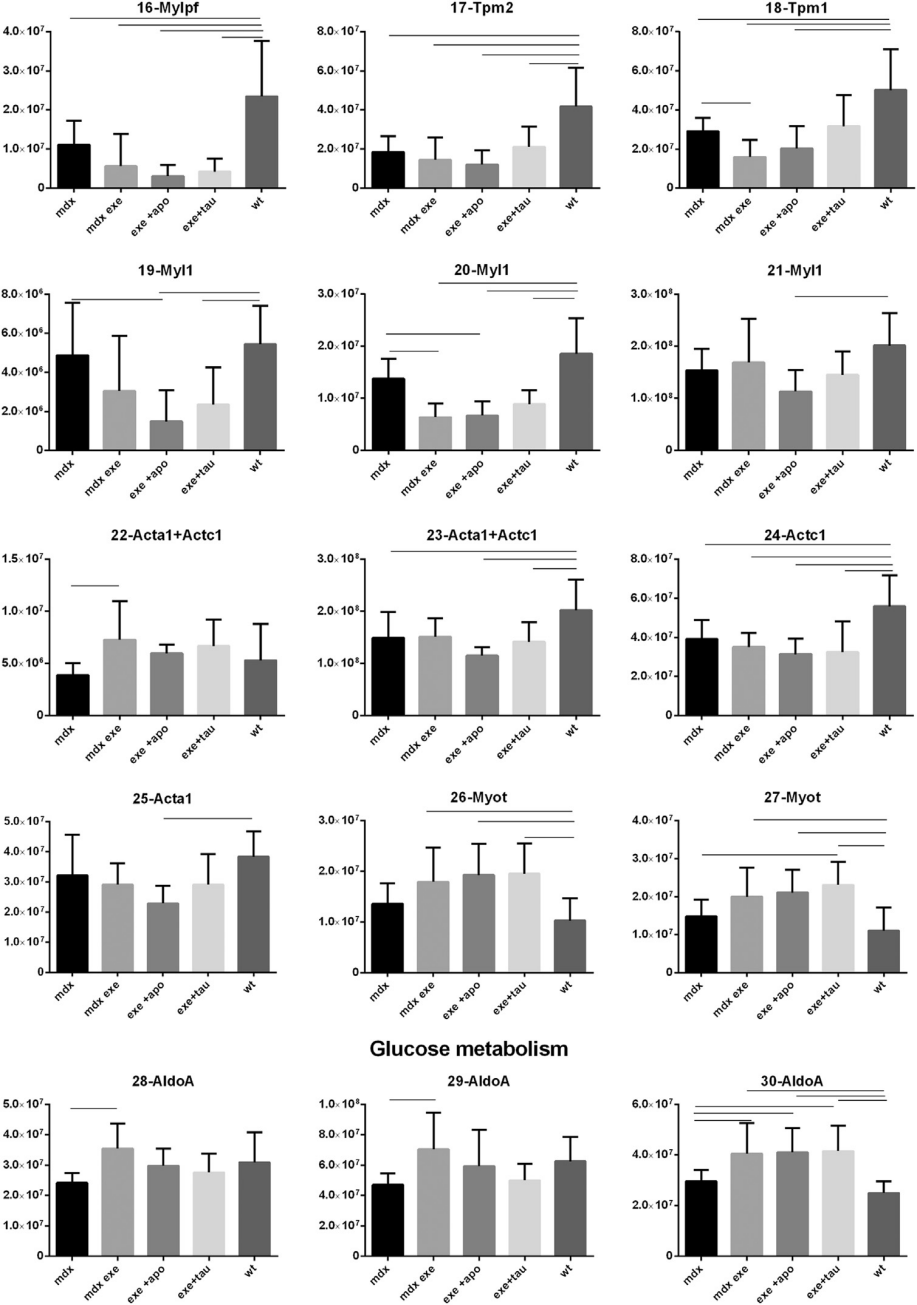

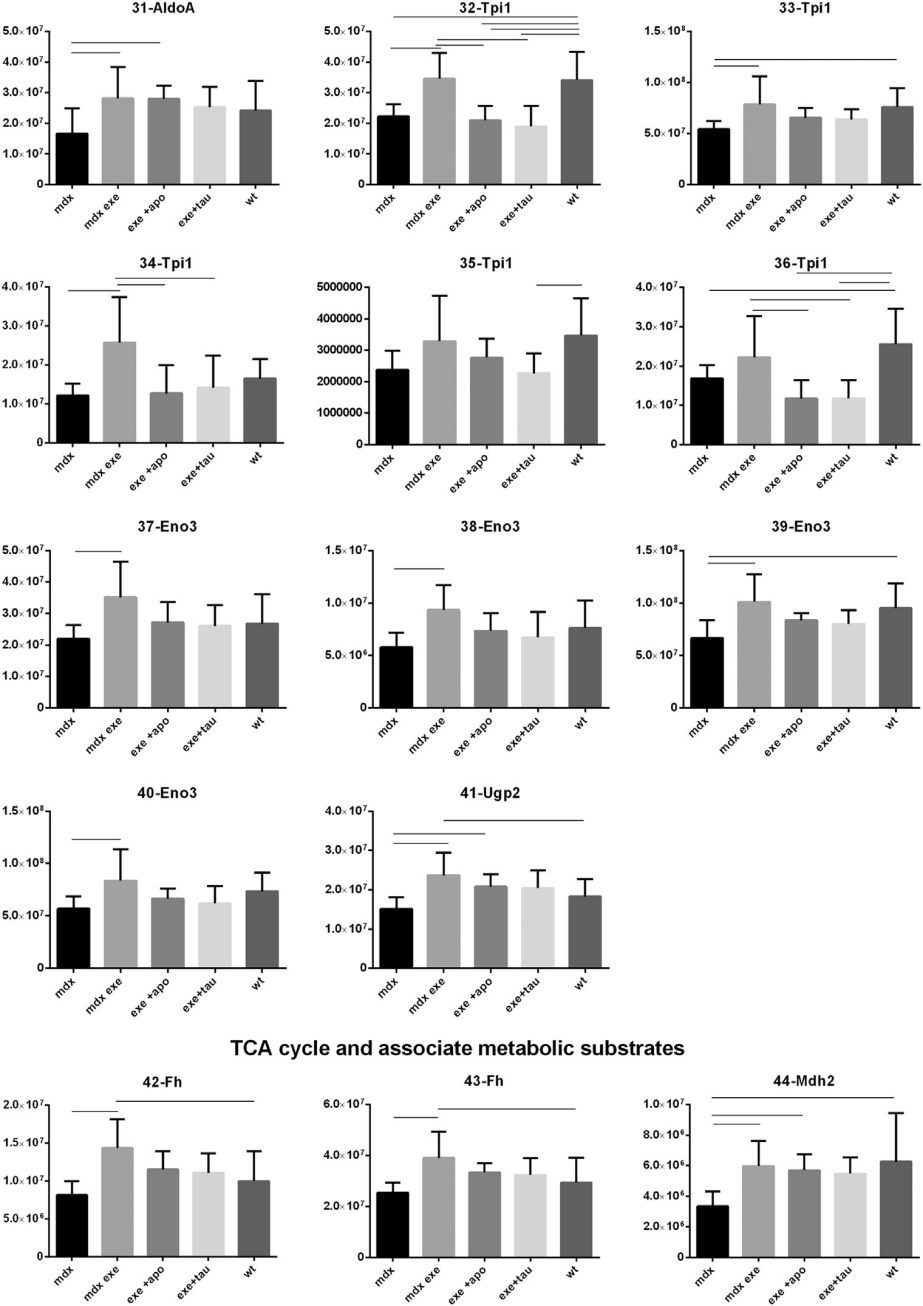

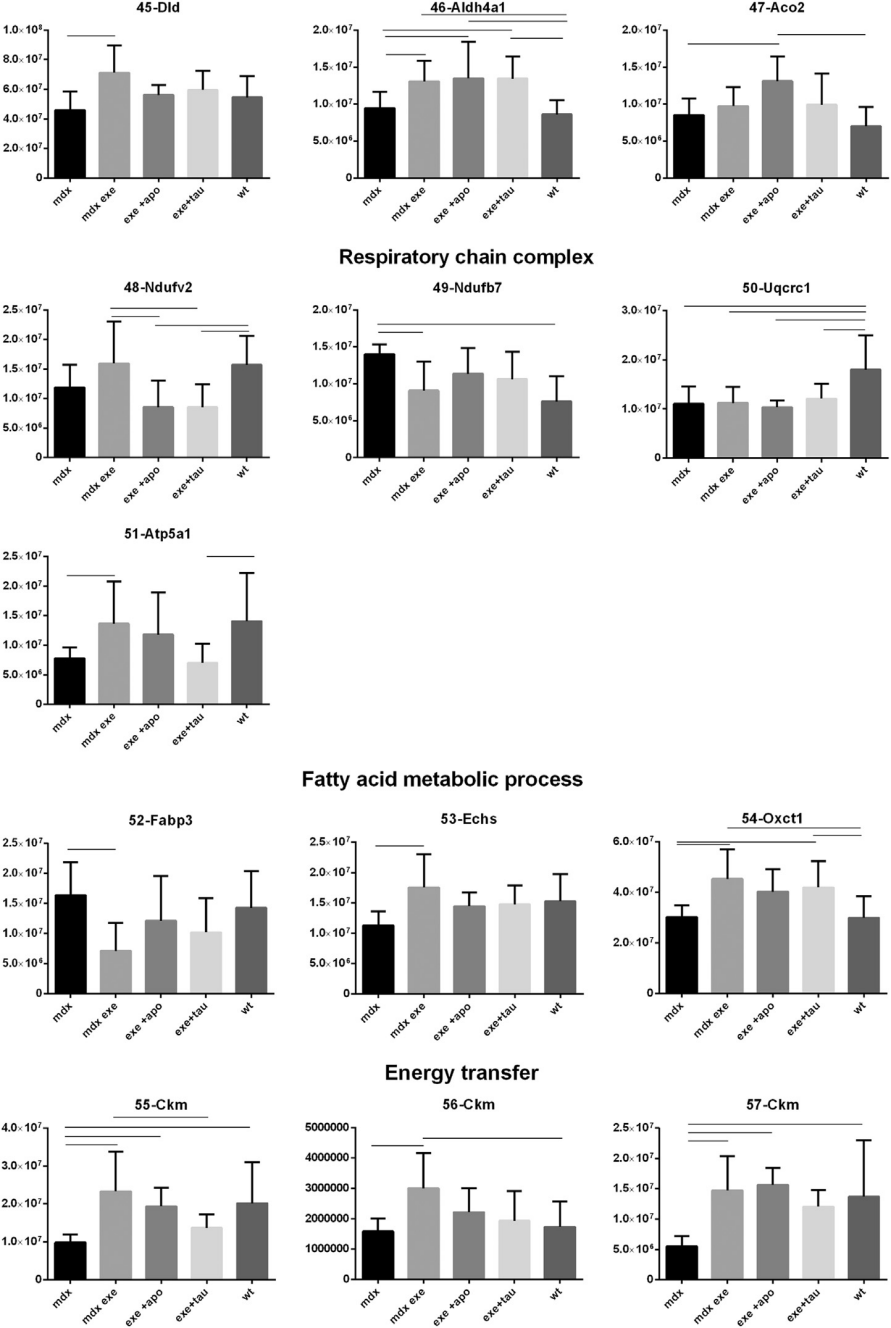

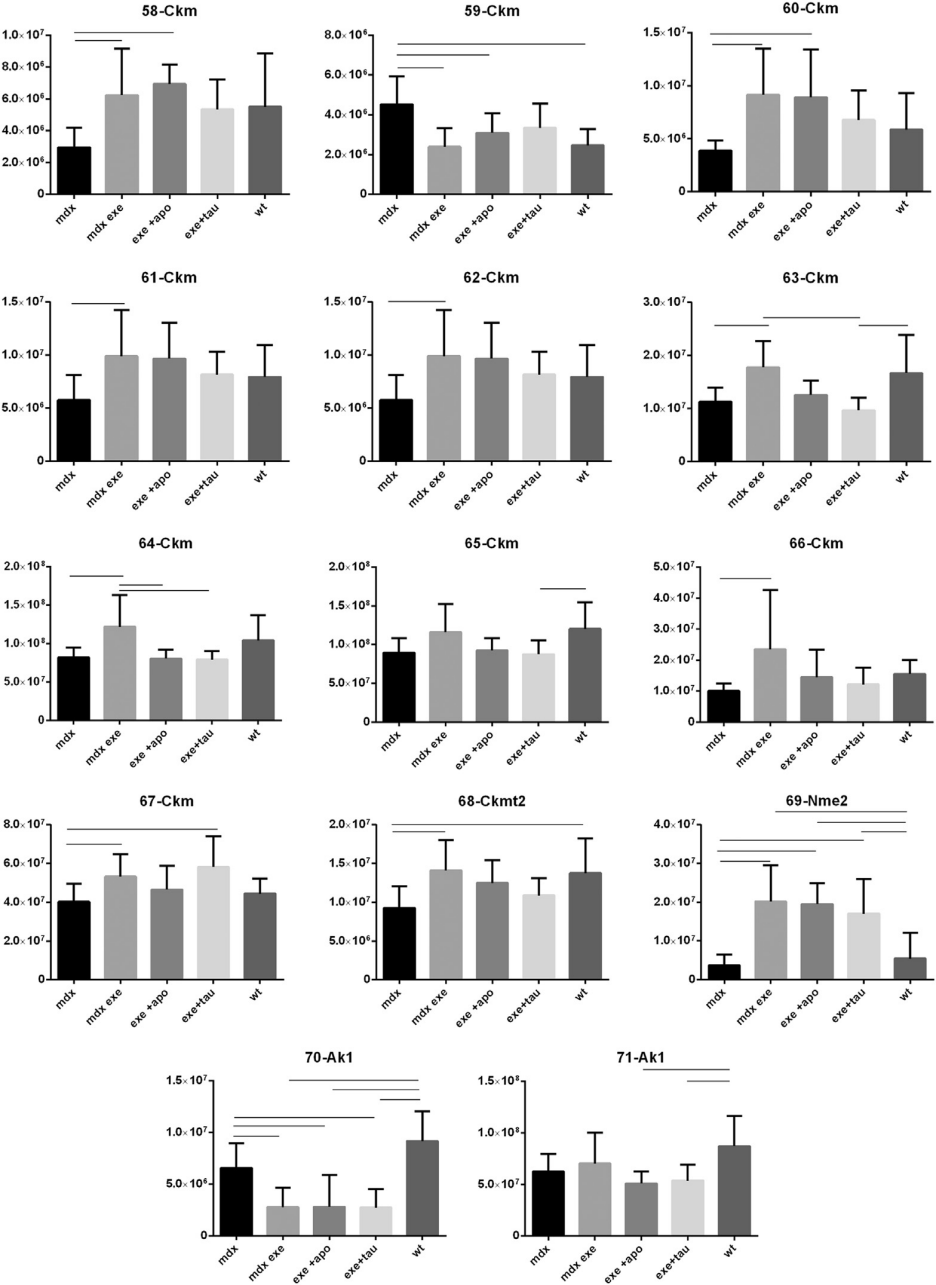

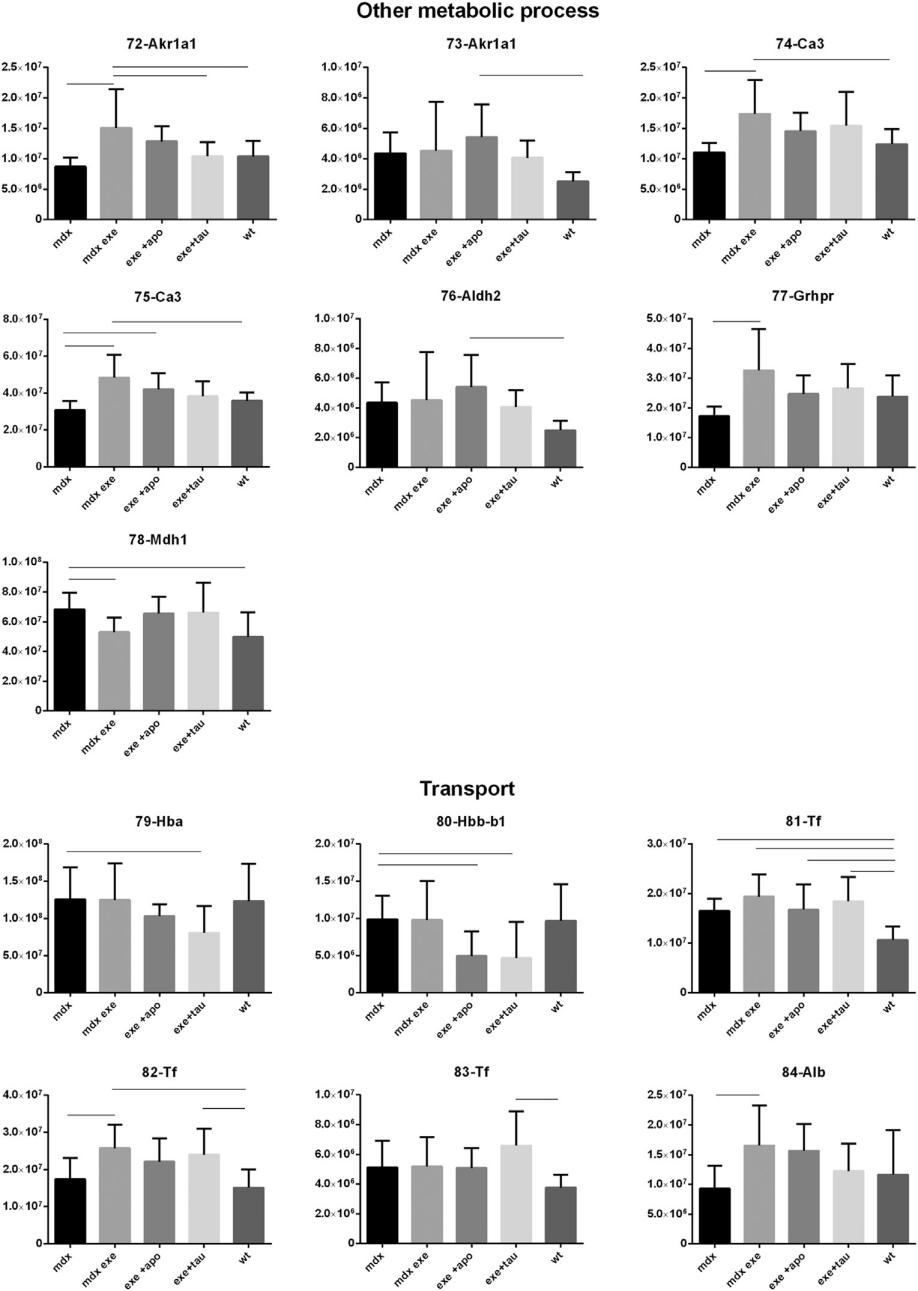

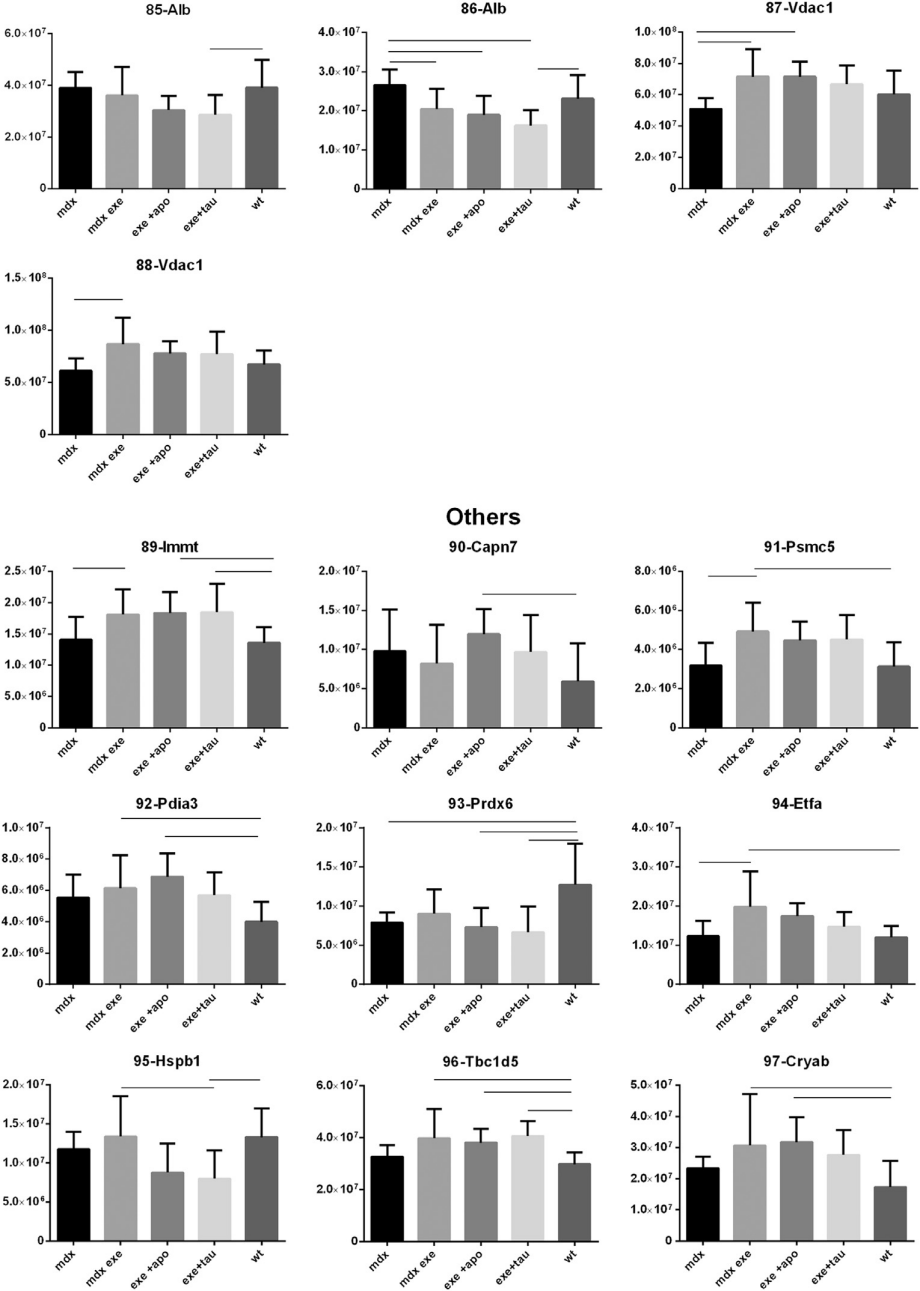
Histograms represent the abundance of each spot (normalized volume, arbitrary units) in all groups studies, namely mdx, mdx exe, mdx exe apo, mdx exe tau (indicated as mdx+apo and mdx+tau respectively) and wt, evaluated with Progenesis SameSpot software. All spots show a False Discovery Rate (FDR) ≤0.05. The significant differences between groups were calculated with GraphPad Prism v6.0 software, using Tukey correction for multiple comparison. Significant differences between groups are indicated by a line.

**Fig. 2:**
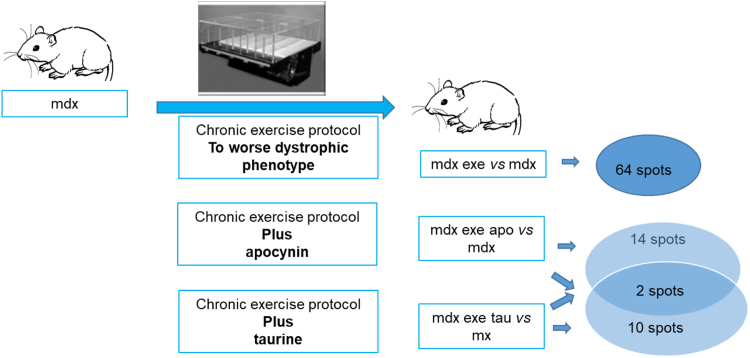
Picture representing different abundant spots between mdx and mdx exe treated and untreated with compounds. Detailed data on spot differences were reported in table 4 of ref [1].

### 1.3 Comparison with wt strain

**Fig. 3:**
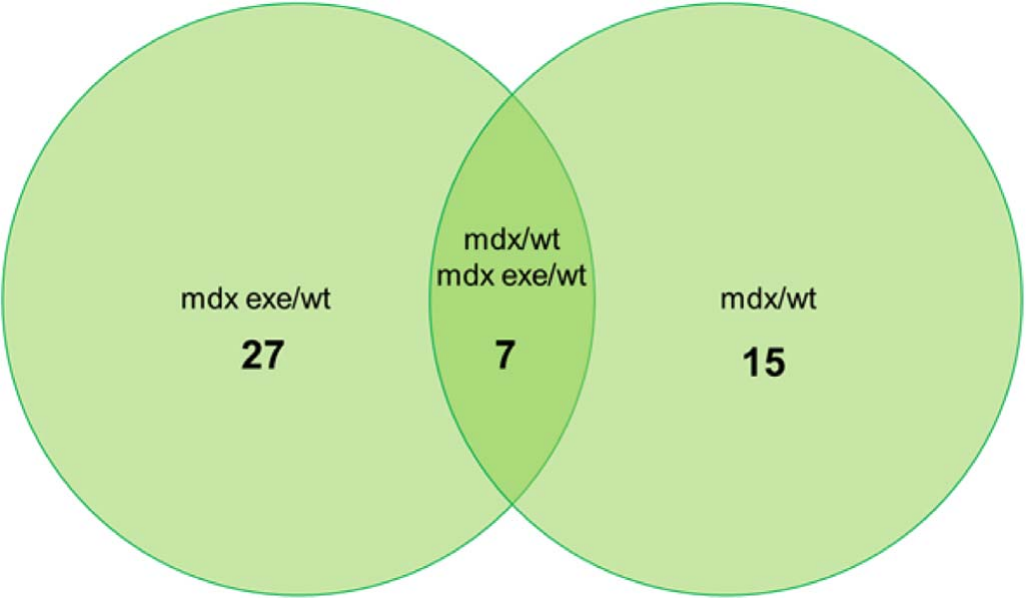
Diagram representing the distribution of differences in spot abundance between groups: 27 protein spots differ exclusively between mdx exe and wt, 15 protein spots differ exclusively between mdx and wt and 7 spots are different from wt in both mdx and mdx exe.

**Fig. 4:**
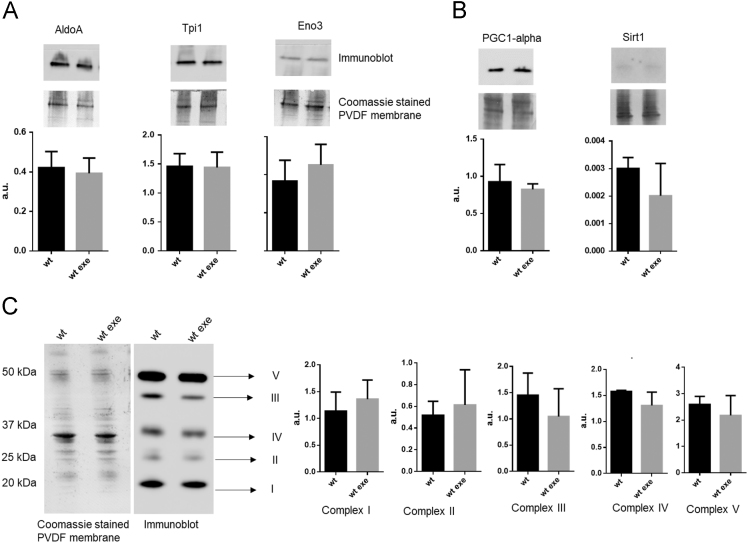
Histograms and representative immunoblot images of glycolytic enzymes: Aldoa, Tpi1 and Eno3 (panel A); PGC1-alpha and Sirt1 (panel B) and Oxphos complexes from wt and wt exe mice. (n=5; mean ± S.D.; t-test unpaired). Normalization of immunoblot was performed on Coomassie stained gel.

Table 3 reports differentially abundant protein spots and relative fold changes, between mdx exe *vs* wt and mdx *vs* wt tibialis anterior muscles. In Fig. 3a diagram represents the relationships between these three groups. The protocol used for mdx training consisted of a 30 min running on a horizontal treadmill (Columbus Instruments, USA) at 12 m/min, twice a week for at least 4 weeks. This protocol causes significant weakness in the limb strength as measured by a grip strength meter [3]. The *in vivo* weakness produced by such a protocol is observed exclusively in mdx mice with no similar effects in wild type mice [4], [5]. In fact, protocols used to induce training effects in wild types mice usually consist of continuous running at 20 m/min for at least 15 min using a treadmill slope of 10°, five days a week, for eight weeks [6]. To exclude training effects in wt animals we checked the amount of selected proteins in wt animals subjected to the same exercise protocol of mdx mice. In particular, we analysed by western blot the amount of several proteins of glycolysis (all increased in mdx exe mice), oxophos proteins, and PGC-1-alpha and Sirt1 proteins. As shown in Fig. 4 none difference is observed in the expression level of these proteins.

**Table 3:** Differentially abundant protein spots between mdx exe *vs* wt and mdx *vs* wt tibialis anterior muscles.

**Spot No**	**Protein name**	a**fold change mdx*****vs*****wt**	a**fold change mdx exe vs wt**
*Sarcomere structure and muscle contraction*			
3	LIM domain-binding protein 3	ns	1.5
4	LIM domain-binding protein 3	ns	1.7
5	LIM domain-binding protein 3	ns	1.8
6	Myozenin-1	ns	1.4
7	Troponin I, fast skeletal muscle	-2.2	ns
8	Troponin I, fast skeletal muscle	-1.6	-1.5
9	Troponin I, fast skeletal muscle	-1.8	ns
14	Myosin regulatory light chain 2, skeletal muscle isoform	ns	-2.1
15	Myosin regulatory light chain 2, skeletal muscle isoform	ns	-3.7
16	Myosin regulatory light chain 2, skeletal muscle isoform	-2.1	-4.1
17	Tropomyosin beta chain	-2.3	-2.8
18	Tropomyosin alpha-1 chain	-1.8	-2.8
20	Myosin light chain 1/3, skeletal muscle isoform	ns	-2.9
23	Actin, alpha skeletal muscle and Actin, alpha cardiac muscle1	-1.4	ns
24	Actin, alpha cardiac muscle 1	-1.4	-1.6
26	Myotilin	ns	1.7
27	Myotilin	ns	1.8
	
*Metabolism and energy transfer*			
30	Fructose-bisphosphate aldolase A	ns	1.6
32	Triosephosphate isomerase	-1.53	ns
33	Triosephosphate isomerase	-1.4	ns
36	Triosephosphate isomerase	-1.52	ns
39	Beta-enolase	-1.4	ns
41	UTP--glucose-1-phosphate uridylyltransferase	ns	1.3
42	Fumarate hydratase, mitochondrial	ns	1.4
43	Fumarate hydratase, mitochondrial	ns	1.3
44	Malate dehydrogenase, mitochondrial	-1.8	ns
46	Delta-1-pyrroline-5-carboxylate dehydrogenase, mitochondrial	ns	1.5
49	NADH dehydrogenase [ubiquinone] 1 beta subcomplex subunit 7	1.8	ns
50	Cytochrome b-c1 complex subunit 1, mitochondrial	-1.6	-1.6
54	Succinyl-CoA:3-ketoacid coenzyme A transferase 1, mitochondrial	ns	1.5
55	Creatine kinase M-type	-2	ns
56	Creatine kinase M-type	ns	1.7
57	Creatine kinase M-type	-2.5	ns
59	Creatine kinase M-type	1.8	ns
68	Creatine kinase M-type	-1.5	ns
69	Nucleoside diphosphate kinase B	ns	3.7
70	Adenylate kinase isoenzyme 1	ns	-2.6
	
*Others*			
72	Alcohol dehydrogenase [NADP(+)]	ns	1.4
74	Carbonic anhydrase 3	ns	1.4
75	Carbonic anhydrase 3	ns	1.3
78	Malate dehydrogenase, cytoplasmic	1.4	ns
81	Serotransferrin	1.5	1.8
82	Serotransferrin	ns	1.7
91	26 S protease regulatory subunit 8	ns	1.6
92	Protein disulfide-isomerase A3	ns	1.5
93	Peroxiredoxin-6	-1.6	ns
94	Electron transfer flavoprotein subunit alpha, mitochondrial	ns	1.4
96	TBC1 domain family member 5	ns	1.3
97	Alpha-crystallin B chain	ns	1.8

a Fold change was calculated dividing the average of %V of mdx or mdx exe by the average of %V of wt (V =volume=integration of the optical density over the spot area; %V = V single spot/V total spots included in the reference gel).

## 2 Experimental design, materials and methods

The methodologies that allowed the data here presented are described in [1] and in cited references. Here, only the protocol for MS/MS data is described.

Trypsin digests of some spots with low Mascot (PMF) score value or with discrepancy between theoretical and calculated MW or pI were further analyzed performing peptide sequencing by tandem mass spectrometry. MS/MS analysis was performed by using an Ultraflex III MALDI- TOF/TOF mass spectrometer (Bruker Daltonics). Two to four PMF peaks showing a high intensity were CID fragmented using Argon as collision gas, and MALDI-TOF/TOF tandem MS was performed in LIFT mode by software controlled data acquisition. Fragmented ions were analyzed using the Flex Analysis software v.3.0. The MS/MS database searching was carried out in the UniProtKB database using the on-line available MASCOT MS/MS ion search software. The following parameters were applied for database searching: taxonomy: *Mus musculus*, trypsin specificity, one missed cleavage allowed, peptide precursor mass tolerance: ±100 ppm, fragment mass tolerance: ±0.6 Da, peptide precursor charge state: +1, carbamidomethylation of cysteine as a fixed modification, oxidation of methionine as a possible modification. For protein identification, Mascot ion score, peptide coverage by “b” and “y” ions, and expected value were considered. We considered as significant, peptides with individual ion scores −10 * Log[P], where P is the probability that the observed match is a random event, that indicated identity (p < 0.05).
